# Medical perspective of reproductive health education in Indonesian schoolbooks

**DOI:** 10.3389/fpubh.2022.943429

**Published:** 2022-11-09

**Authors:** Wienta Diarsvitri, Iwu Dwisetyani Utomo

**Affiliations:** ^1^Department of Community Medicine, Faculty of Medicine, Hang Tuah University, Surabaya, Indonesia; ^2^School of Demography, College of Arts and Social Sciences, Australian National University, Canberra, ACT, Australia

**Keywords:** medical perspective, reproductive health education, Indonesia, schoolbooks, inaccurate information

## Abstract

The current provision for equipping young Indonesians with a comprehensive knowledge of reproductive health is inadequate. In Indonesian primary and secondary schools, reproductive health education is integrated into various subjects, including Science, Biology, Sport, and Health Education. In this paper, we compared the accuracy of the material related to reproductive health education to scientific evidence published in medical scientific journals or medical textbooks. Even though the schoolbooks were used in Indonesia's 2006 minimum standard requirements of subject matter (KTSP) curriculum, we found much inaccurate information that is not based on the scientific literature and unnecessarily detailed information on therapy and technology. Schoolbooks should emphasize promoting a healthy lifestyle, preventing high-risk sexual behaviors, encouraging openness and discussion about reproductive health in the family, improving self-confidence to refuse and avoid sexual harassment, encouraging positive sexual behaviors, and increasing awareness for treatment-seeking behavior.

## Introduction

Studies conducted on more than 19,000 never-married women and men aged 15–24 years in all 33 provinces of Indonesia revealed that young Indonesians had not had enough knowledge of reproductive health. Overall, less than 60 percent of respondents completely understood physical changes at puberty. Less than 26 percent of male respondents knew that a woman's fertile period is halfway between periods. Less than 56 percent of respondents knew that a woman can become pregnant after one instance of unprotected sexual intercourse (1, 2).

A study on 1,762 year 6 primary school students in four provinces in Indonesia showed variability in the student's understanding of how pregnancy can occur (3). In addition, a study conducted on 1,082 senior high school students in Papua and West Papua Provinces found that around 38.3 percent of students reported having had sexual intercourse, and 36.5 percent of them had had their first sexual intercourse before they were 15 years old (4).

Below is the confession of a young Indonesian female who got pregnant when she was 15 years old and still in her first year at a high school in Bandung, West Java. To terminate her pregnancy, her boyfriend asked her to drink grated young pineapple mixed with soda drinks and arak (homemade distilled alcohol). In addition, she also consumed large amounts of herbal medicines to abort the fetus. Despite her efforts, her pregnancy got more extensive, and with her parent's support, she finally gave birth to a healthy baby boy (5).

“*I had been grappling with feelings of guilt, shame, isolation, and desperation for years. If I had been able to get information and access to information on reproductive health and youth-friendly health services earlier, my life would have been different,”* said Nindya (not her real name), who is now an active youth advocate and a peer educator (5).

The formal education system in Indonesia consists of early childhood education; primary, secondary, and tertiary level. a 12-year compulsory education in primary (6 years) and secondary Indonesia, consisting of 6 years of primary school, 3 years of junior high school, and 3 years of senior high school. In 2006, the Indonesian government introduced the education unit level curriculum (*KTSP*) for elementary, junior high, and senior high schools. *KTSP* was a decentralized school-based curriculum, a replacement of the earlier curriculum called competency-based curriculum (*KBK*). In the *KTSP* system that could be used until 2020, the government acknowledged the diversity of local context, therefore each provincial or district government might design its own curriculum based on its context, as well as referring to curriculum policy and guidelines issued by the National Education Standard Board (NESB) (6). With increasing net enrollment ratios of primary to senior high schools in Indonesia (7), formal education has an excellent opportunity to reach large numbers of young people. Moreover, schoolbooks used in formal education have significant contributions to preparing young people with essential knowledge, attitude, and skills needed to make the responsible choice of healthy behavior and sexual practices.

This study presented the medical perspective of reproductive health education in the primary and secondary schoolbooks used in Indonesia's 2006 *KTSP* curriculum. In the *KTSP* curriculum, reproductive health education in Indonesia was integrated into four compulsory related subject matters: Biology (Science), Social Science *(IPS)*, Religion, and Sport and Health Education *(Penjaskes)* (8). The analysis was part of the 2008 Indonesian Gender and Reproductive Health Book Analysis Study.

## Methods

This study adopted a qualitative approach by employing a literature review and content analysis (9, 10). In the data collection step, descriptions or vital words relating directly or indirectly to reproductive health were recorded. Books corresponding to elementary, junior high, and senior high school grades, as well as subjects from various publishers, were purchased. In the data analysis step, a total of 172 books of Biology (Science), Social Science *(IPS)*, Religion, and Sport and Health Education *(Penjaskes)* produced by more than 15 publishers were intensively analyzed. We compared the accuracy of the contents in the books to scientific evidence-based literature published in medical scientific journals or medical textbooks. In this article, we presented the analysis of 51 schoolbooks of years 5-12, consisting of 23 biology and science books and 28 sports and health education books. This study covered four areas of reproductive health: genital hygiene, sexually transmitted infections (STIs), pregnancy and delivery, violence, and sexual crimes.

## Results

### Genital hygiene

In general, some schoolbooks provided inaccurate information related to the anatomy and physiology of urinary and reproductive systems, as well as genital hygiene, as depicted in Table 1.

**Table 1:** Some inaccurate information about genital hygiene.

**Year and book**	**Inaccurate information**
Year 5 sport and health education *(Penjaskes)* Books	1. Reproductive organs are external genitals. Clean genital after urination and defecation with clean water [(11), p. 47]. 2. Reproductive organs can be infected because they also serve as urinary tracts. Testis functions to get the urine out from the bladder. The vagina functions as an organ to get offspring and a urinary tract [(12), p. 61].
Year 6 science book	Genital should be washed after urination. Some ways to keep female genital hygiene include regular exercise. [(13), p. 18].

Some schoolbooks provided overlapped understanding related to reproductive and urinary systems, which are two different biological systems of human bodies that carry out specific functions. In females, the urethra is the end part of the urinary system, and it is not connected to the vagina. In males, the urethra extends from the urinary bladder to the distal end of the penis, and the testis is not part of the urinary system. The urethra is the passageway for both urine and male reproductive fluids. The two, however, do not exit the urethra at the same time. While seminal fluid passes through the urethra, a reflex causes the urinary sphincter muscles to contract tightly to keep urine from passing the urinary bladder through the urethra (14).

One of the female genital hygiene practices that are important for schoolgirls but are not covered in reproductive health education at schools in Indonesia is menstrual hygiene. Lack of adequate knowledge and misbelieves related to menstruation were found in a study conducted on 1,402 adolescent schoolgirls aged 12-19 years from 16 schools in four provinces of Indonesia (East Java, South Sulawesi, Papua, and East Nusa Tenggara). Around 97 percent of girls reported that they had heard about menstruation before menarche; however, many stated they were still unprepared and confused when they first menstruated and lacked adequate knowledge to deal with menstruation. Misbeliefs surrounding menstruation was still common among girls in the study, including they could not do physical activity during menstruation, they should avoid particular food during menstruation, menstrual blood contained dangerous substances, a girl was dirty or unclean during menstruation, and washing hair during menstruation could block the menstrual flow, caused headache or death (15).

### Sexually transmitted infections

Much of the inaccurate information in the year 7, 9, and 11 books were about the cause and transmission of syphilis, HIV, and trachoma, as depicted in Table 2.

**Table 2:** Some inaccurate information about STIs.

**Year and book**	**Inaccurate information**
Year 7 Sport and Health Education *(Penjaskes)* Books	1. The cause of STIs is a virus that emerges because of infection (acute wound) on the human genital [(16), p. 142]. 2. Blindness (trachoma) is caused by vitamin A deficiency [(17), p. 93]. 3. STIs can only be transmitted *via* sexual relationships (sexual organs) [(17), p. 176]. 4. STI prevention includes exercise and a healthy diet [(17), p. 179]. 5. STIs induce infection on several organs, including the digestive tract and liver [(18), p. 237]. 6. Syphilis can be transmitted by contact with contaminated vomit [(18), p. 240]. 7. Painful on the hip bone and inflammation on the hip bone are gonorrhea's symptoms [(18), p. 241]. 8. Untreated STIs will cause chronic diarrhea [(19), p. 226]
	9. Syphilis causes painful ulceration called a chancre. The pain is felt around the genitals, mouth, tongue, and other body parts [(19), p. 227]. 10. Husband and wife should use a contraception device when having sexual intercourse [(19), p. 230].
Year 11 sport and health education *(Penjaskes)* books	1. CIV (Ciuman Immunodeficiency Virus) was known to be identical with HIV (Human Immunodeficiency Virus) [(20), p. 106]. 2. HIV attacks the red blood cells [(20), p. 107]. 3. HIV/AIDS can be prevented by being aware of safe sex (sexual contact without mixing genital fluid as a way of HIV transmission) [(20), p. 108]. 4. Some ways to prevent HIV infection include avoiding stress (21).
Year 9 science books	1. Gonorrhea and syphilis are caused by fungi [(22), p. 23].
Year 11 biology book	1. Symptoms of STIs in men: severe itching and genital, hot, swelling, and pain on the hip, which then become ulcers. Symptoms of STIs in women: spotting after sexual intercourse [(23), p. 314]. 2. There are five stages of syphilis. The fifth stage is congenital syphilis [(24), p. 216]. 3. Herpes simplex is caused by the Varicella zoster virus [(25), p. 223].

Regarding sexually transmitted infections (STIs), it should be explained that T. pallidum, a spirochaete bacterium, causes syphilis. The disease may be congenital or acquired. Congenital syphilis is transmitted from mother to child during pregnancy. Acquired syphilis is classified as early syphilis and late syphilis. Primary syphilis, the first stage of early syphilis, is characterized by the occurrence of a sore (called a chancre) that is usually firm, round, small, and painless. It appears at the spot where syphilis entered the body. Regarding HIV, the virus attacks the immune system, specifically the CD4-T cells, part of the white blood cells. About trachoma, it is the world's leading cause of preventable blindness caused by *Chlamydia trachomatis*, the same bacterium that causes Chlamydiosis, a common sexually transmitted infection among teenagers (26).

Some year six books explained that around 53 percent of HIV/AIDS transmission in Indonesia is due to sharing needles by drug users (27), which was not valid. From 1987 to March 2017, the majority (68 percent) of HIV cases in Indonesia were transmitted by heterosexual contact, followed by blood transfusion (13 percent), and sharing needles among injecting drug users (11 percent). Moreover, most HIV-infected people in Indonesia were from 25-49 and 20-24 age groups (28), who were sexually active.

Some schoolbooks gave detailed information on gonorrhea therapy, such as ceftriaxone, cefixime, ciprofloxacin, and ofloxacin (16). Other books explained antiretroviral treatment (ART) for HIV/AIDS globally, their composition, how they work, and their side effects (20). It is enough that elementary to senior high school students know there is an available treatment for STIs, but they do not have to know the type and name of treatment or even the mechanism of how a particular drug works.

**Figure 1:**
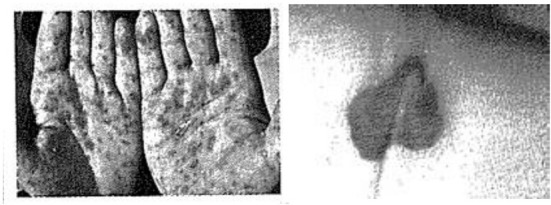
Rash on the palm **(left)** as a symptom of syphilis (29), and inflammation on the skin **(right)** (18) as a symptom of HIV Infection.

Further, few schoolbooks in Indonesia provided photographs of STIs, but the pictures showed the effect of STIs on the skin. Gonorrhea was depicted as congenital blindness; the symptom of syphilis was shown as a rash on the palm or plantar surface. AIDS was often depicted as having a rash on the skin, as depicted in Figure 1 (18). The use of photographs showing the effect of STIs on the skin may create a misperception that STIs are skin diseases and have minimal impact on sexual practices. Further, students who read the non-explicit materials may not perceive a threat, susceptibility, and severity, as highlighted in the Health Belief Model (30). Due to the sensitive nature of explicit material, an essential element of any plan related to this matter in Indonesian schools must also consider the importance of teachers' training so that they feel comfortable teaching the materials (31).

The critical messages related to STIs that should be delivered to the schoolers, for example, are: (1) STIs can be transmitted in various ways, and not exclusively through sexual intercourse. Early diagnosis and prompt treatment are needed to prevent serious complications. (26); (2) The ABC of prevention should be known by students with abstinence as the best choice to prevent STIs. It has to be emphasized that using a condom in ABC prevention is regarded as “safer sex,” but not “safe sex” (32), so teenagers are not encouraged to have sexual relations that seem safe and problem-free; (3) An effective prevention strategy across multiple sectors should be implemented, including the health care system, educational, social, and policy (33).

### Pregnancy and delivery

Some schoolbooks for years 6, 9, and 11 gave inaccurate information about fetal growth during pregnancy and the delivery process, as depicted in Table 3.

**Table 3:** Some inaccurate information about pregnancy and delivery.

**Year and book**	**Inaccurate information**
Year 6 science book	A baby will be born by external pushing and contraction of the uterus [(13), p. 19].
Year 8 science book	1. Placenta and umbilical cord will be cut during delivery [(34), p. 9].
	2. The first stage of delivery is dilation of the cervix, and the second stage is a contraction of the uterus [(35), p. 13].
Year 9 science book	The delivery process starts with dilation of the cervix, followed by contraction of the uterus [(36), p. 26].
Year 11 biology book	In the ninth month, the length of the fetus is 950 mm = 95 cm [(25), pp. 217–218].

Pregnancy is, for most women, a time of great happiness. However, during pregnancy, both the woman and her developing fetus face various health risks. Therefore, the message that should be delivered to the students is the importance of all pregnancies to be well prepared and monitored by skilled care providers. To achieve a healthy pregnancy, an expectant mother should have a good nutritional status, proper antenatal care, regular physical activity, preventive measures against various infections, and an excellent psychological condition (37).

Labor is the physiologic process by which a fetus is expelled from the uterus. Normal labor is divided into three stages. In the first stage, biochemical connective tissue changes in the cervix precede progressive rhythmic uterine contractions and lead to cervical dilatation. All of these events culminate in spontaneous rupture of the fetal membranes. The second stage of labor is the time between complete cervical dilation and delivery of the neonate. The delivery of the placenta is the third and final stage of labor; it usually occurs within 30 min of delivery of the newborn (38). The length of the fetus in the ninth month of pregnancy is around 48-53 cm, in opposition to the size of 95 cm written in the year 11 Biology book (25).

The second stage of labor comprises the passive phase, with passive descent of the fetal head, and the active phase or expulsive phase, which starts when contraction becomes expulsive or when the pregnant mother actively pushes. Optimal obstetric management of the second stage is an ongoing challenge to reduce emergency cesarean delivery rates and avoid adverse maternal and neonatal outcomes (39). The fundal pressure (Kristeller maneuver), an application of external manual pressure to assist spontaneous vaginal delivery during the second stage of labor, is a common practice conducted at home by birth attendants or at hospitals by health workers, even though the course is often not documented in the medical record (40). Contrary to the written explanation in the schoolbook (13), the maneuver was ineffective in shortening the second stage of labor and added riskier than beneficial, which leads to vaginal, cervical, and third-degree perineal laceration; uterine prolapse, retained placenta, postpartum hemorrhage, increases the rate of episiotomy and Cesarean delivery (41).

Many Indonesian female teenagers did not know that they could be pregnant after having one sexual intercourse (1, 2). The withdrawal method is not a safe method for pregnancy prevention. Most female teenagers are not ready to become pregnant. Students should know that pregnancy could happen when a girl reaches puberty and has sex with a man who can produce sperm. However, reaching puberty does not mean that a girl is ready for pregnancy. Complications from pregnancy and childbirth are the leading cause of death among adolescent girls aged 15–19 years in low and middle-income countries. Infants of adolescent mothers are more likely to have a preterm delivery, low birth weight, and severe neonatal condition, which can long-term impact their health and development. Therefore, students should avoid having sex at a young age (42).

High school students should know that Indonesia is one of the high-burden countries related to maternal mortality (43). To reduce the maternal mortality rate, Indonesia had several efforts, including increasing services in some public health centers (*Puskesmas*) as primary health care service providers to provide essential obstetric and neonatology emergency services (PONED) (44), and in hospitals as a secondary health care provider to provide comprehensive obstetric and neonatology emergency services (PONEK) that are available for 24 h (45).

However, students should be aware that there are many potential barriers to accessing emergency obstetric care that is conceptualized in the “three delays model” of Thaddeus and Maine: delays in (i) deciding to seek care; (ii) reaching a health facility; and (iii) receiving appropriate health care (46, 47). Either a single delay or a sequence of delays can be fatal. There are also common causes of high-risk maternal mortality that should be recognized in the community: too old for having a child, too young for having a child, too many children, and too close spacing of each child. Public health efforts to reduce maternal mortality in Indonesia include the use of a mother and child health book (*Buku KIA*) for every pregnant mother, as well as delivery planning and prevention of complication stickers (P4K sticker) that will be put on the house door of every high-risk expectant mother. The sticker is meant to let the neighborhood know about the presence of high-risk pregnancy mothers, so they are ready to help whenever needed (48).

In addition, some year 8-11 schoolbooks explained various techniques used in reproductive technology, including *in vitro* fertilization (IVF), partial zona dissection (PZD), subzonal sperm intersection (SUZI), and intra cytoplasm sperm injection (ISIS). The books also explained about two techniques used to obtain sperm, including microsurgical sperm aspiration (MESA) and testicular sperm extraction (TESE) (36, 49). Do high school students need to know the detail of the sophisticated technology of *in vitro* fertilization?

### Violence and sexual crime

Some schoolbooks gave inaccurate information about the causes of violence and sexual crimes, as depicted in Table 4.

**Table 4:** Some inaccurate information about violence and sexual crimes.

**Year and book**	**Inaccurate information**
Year 5 sport and health education *(Penjaskes)* books	1. Going out with an adult can lead to sexual harassment [(50), p. 68]. 2. Some physical signs of sexual harassment are urinary tract inflammation and throat infection. Some non-physical signs of sexual harassment are growth retardation and isolation [(12), p. 62].
	3. The victims of sexual harassment are women and men under 18 years old [(12), p. 63].
Year 6 sport and health education *(Penjaskes)* books	1. Children should not allow other people to see or touch their bodies to avoid sexual harassment [(51), p. 124]. 2. Students should avoid sexual harassment by choosing a seat beside the window when taking public transportation [(50), p. 136].
	3. Victims of sexual harassment are boys and girls under 18 years old [(27), p. 143].
	4. Female students should wear decent clothes and avoid “excessive” cosmetics to prevent sexual harassment [(27), p. 142].

Violence is “the intentional use of physical force or power, threatened or actual, against oneself, or a group or community that either result in or have a high likelihood of resulting in injury, death, psychological harm, maldevelopment, or deprivation” (52). Violence-related problems can be depicted as a pyramid, with violent death representing the pyramid's apex, the victims that need care in the middle of the pyramid, and acts of violence that are often not reported are in the bottom (53).

Indonesia has ratified the United Nations Convention on the Rights of the Child (UNCRC) 1989 (54) and published a Presidential Decree No. 36/1990 (55). Thirteen years after the launch of UNCRC, Indonesia issued Law No. 23/2002 on the protection of the child (56), which was followed by the establishment of the Indonesian Child Protection Commission (KPAI) as one of three independent national institutions that guard the implementation of human rights in Indonesia. In 2014, Law No. 23/2002 was revised by Law No. 35/2014 to protect the child (57). Yet, there have been weaknesses and gaps in protection afforded to children against all forms of violence.

A survey conducted in 25 provinces, 108 districts, and 125 subdistricts revealed that 47.45 percent of men (1 of 2 men) and 35.05 percent of women (1 of 3 women) aged 18-24 years reported having had sexual, physical, and emotional violence (s) when they were < 18 years. Approximately 30 percent of boys and girls aged 13-17 years reported having had sexual, physical, and emotional violence (s) in the last 12 months. However, the majority of those who experienced violence (62.6 percent of men and 86.87 percent of women aged 18-24; and 75 percent of boys and 85.4 percent of girls aged 13–17 years) did not know any available child protection services and did not ask for help when they experienced violence (58). As of 2015, the KPAI received 289 reported cases related to children as victims of trafficking (48 cases), online prostitution (96 points), commercial sex (61 patients), and child labor (84 cases) (40b). Despite the available protection, children should have knowledge and skills on preventing sexual violence, including tips for partying, avoidance strategies, and crime prevention tips (59–61).

Related to body-revealing cloth, several year 5 and 6 Sport and Health Education books reminded female students to wear ‘decent' clothes as a preventive measure against sexual harassment. The information in these books implied a gender bias. Further, there could be various interpretations of the term “decent” clothes, depending on the different cultural contexts. Of five books that suggested students wear decent clothes, three books explained proper meaning. One book described decent clothes as clothes that accentuated the body shape. This book also prohibited female students from using “excessive” cosmetics (27). Another book suggested that students should not wear short skirts or tight clothes (62). Another book described decent clothes as clothes that did not show the thighs, breasts, or bellies (63). These suggestions on female clothing were written in sections on preventing sexual harassment.

A study explored the reasons for the allegation that female victims of sexual violence precipitate their assaults by wearing provocative, body-revealing clothes. The survey of 193 female and 128 male undergraduate students aged 18–24 years revealed a gender-based attribution gap. Men perceived the sexualized look as indicating an interest in sex and intent to seduce. Women reported wearing sexy clothes to feel and look attractive (64). Women's attractiveness was also connected with color, and a study found that men reported higher sexual intent in women wearing red clothing (65).

## Discussion

This study found much inaccurate information related to genital hygiene, STIs, pregnancy, delivery, and sexual violence in the school books of primary, junior high, and senior high school books. Belief in inaccurate or false information may lead to poor judgments, decision-making, and an enduring impact on people's thoughts that results in resistance to correction (66). Accurate and reproducible SRH knowledge is crucial because it may facilitate students' decision-making process. The process may be explained by the “mindsponge” theory, “a metaphor that the mind is analogized to a sponge that absorbs new compatible values and squeezes out incompatible values with its core values.” The core values are highly trusted values or beliefs, that are used as points of reference for assessing the suitability of newly absorbed values (or information) that influences an individual's perceptions, attitudes, and behaviors, as well as making decisions or responses [(67), p. 5].

Prior studies have examined the impact of health literacy and reproductive health education on students and the results showed that students receiving reproductive health education were more responsible for their sexual practices (68). High-quality schoolbooks are an essential part of learning materials and are intended as one of the students' primary sources of information. If left to the students' own devices to find information and draw up their own perception of reproductive health, adolescents may have poor knowledge of reproductive health and may be at risk of practicing unsafe sex (69). Therefore, the schoolbooks” crucial health information and skills must be scientific-based and reliable (70).

At the 1994 International Conference on Population and Development (ICPD), governments from 179 countries including Indonesia affirmed that adolescents need and have a fundamental right to obtain sexual and reproductive health (SRH) information and services to help them develop positive values, attitudes, and informed decision practices (71). Prior studies reported that the majority of school students in Indonesia obtained SRH knowledge from the media (4, 72). As of 2021, the timely school participation rate in elementary, junior high, and senior high schools in Indonesia were 97.8%, 80.6, and 61.7%, respectively. These data were inclusive of female and male school-age children, in urban and rural areas, and children with and without special needs (73). As the majority of children and youth in Indonesia attend formal education, schools have an important role to promote SRH as part of fundamental aspects of life. Therefore, we recommend that schoolbooks should provide age-appropriate, accurate, culturally relevant, and non-judgmental SRH information, for example, related to genital hygiene, high-risk sexual behaviors, how pregnancy can occur; as well as putting emphasis on the prevention of risky sexual behaviors, STIs, reproductive health problems, and sexual violence in adolescence that may have long-lasting consequences. Moreover, the development of schoolbooks that contain SRH materials can be carried out in collaboration with university lecturers from medical or health-related schools, based on the scientific references, such as medical textbooks or journals.

The advancement of information technology allows anyone to access various information, including SRH. To prevent misinformation, we recommend that scientific-based SRH information should be made available and can be accessed by vulnerable or marginalized adolescents, those who cannot attend formal education, and the community. In addition to scientific-based SRH information, effective SRH services should also be available for adolescents (74). In Indonesia, youth-friendly healthcare facilities at primary and secondary levels, as well as school-based healthcare services and various community-based health programs for adolescence are available, but their quality should be improved.

## Conclusion

The current provision for equipping young Indonesians with a comprehensive knowledge of reproductive health is inadequate. The existing reproductive health materials in the primary to senior high schoolbooks contain incomplete and inaccurate information in the schoolbooks that are not based on the scientific literature and are presented in a complex language. They have unnecessarily detailed information on the therapy and technology. Primary to high school students do not need thorough treatment for HIV and other sexually transmitted infections and detailed assisted reproductive technology such as medical students. Moreover, some schoolbooks explain the harmful use of external pushing in delivery and lead to many adverse effects.

Students have the right to obtain all the relevant and scientifically grounded information on reproductive health to decide their steps. During the review, we found that many references in the schoolbooks were from personal blogs or websites that were not based on scientific evidence. We recommend that the contents and illustrations of the books should be based on scientific evidence, such as articles in scientific journals or textbooks. The schoolbooks should emphasize promoting a healthy lifestyle; preventing high-risk sexual behaviors (i.e., early sexual debut, having multiple sexual partners, using substance abuse); encouraging openness and discussion about reproductive health in the family, improving self-confidence to refuse and avoid sexual harassment, encouraging positive sexual behaviors, and increasing awareness for treatment-seeking behavior.

## Data availability statement

The original contributions presented in the study are included in the article/supplementary material, further inquiries can be directed to the corresponding author.

## Author contributions

IDU designed and directed the project. WD performed the analysis and drafted the manuscript. WD and IDU discussed the results and commented on the manuscript. Both authors contributed to the article and approved the submitted version.

## Funding

This research was made possible by funding from the AusAID through the Australian Development Research Award, Ford Foundation, ADSRI-ANU, the Indonesian National Planning Bureau-BAPPENAS obtained by IDU, and the 2018 Endeavour Executive Fellowship obtained by WD. The publication was made possible by funding from the Faculty of Medicine, Hang Tuah University, Surabaya, Indonesia.

## Conflict of interest

The authors declare that the research was conducted in the absence of any commercial or financial relationships that could be construed as a potential conflict of interest.

## Publisher's note

All claims expressed in this article are solely those of the authors and do not necessarily represent those of their affiliated organizations, or those of the publisher, the editors and the reviewers. Any product that may be evaluated in this article, or claim that may be made by its manufacturer, is not guaranteed or endorsed by the publisher.
